# Supplementary material for: Turbidity interferes with foraging success of visual but not chemosensory predators
Source: PeerJ. 2015 Sep 8;3:e1212. doi: 10.7717/peerj.1212 (PMC4579029; doi:10.7717/peerj.1212)
Supplement: Supplemental Information 1 [file peerj-03-1212-s001.pdf]

ERIN L. SHERMAN, MAcc, CRA, CIP, CPIA
Research Compliance Officer
Division of Research, Commercialization and Outreach

6300 Ocean Drive, Unit 5844
Corpus Christi, Texas 78412
O 361.825.2497 • F 361.825.2755

Institutional Animal Care and Use Committee (IACUC)

APPROVAL DATE: February 8, 2013

TO: Dr. Lee Smee

CC:

FROM: Institutional Animal Care and Use Committee (IACUC)
Office of Research Compliance

SUBJECT: Initial Approval

Protocol Number: 07-07

Title: Various invertebrate experiments

Review Category: Invertebrate

This research project has been approved. As principal investigator, you assume the following responsibilities:

1. If animals are housed on campus, a copy of this approval letter should be kept in the lab notebook the IACUC will review during its semiannual facility inspections.
2. Amendments: Changes to the protocol must be requested by submitting an Amendment Application to the Research Compliance Office for review. The Amendment must be approved before being implemented.
3. Completion Report: Upon completion of the research project (including data analysis and final written papers), a Completion Report must be submitted to the Research Compliance Office.
4. Records Retention: Records must be retained for three years beyond the completion date of the study.

Provisions:

Comments: The IACUC voted to modify invertebrate procedures. Invertebrate animal use protocols will no longer have an expiration date. Please submit an amendment if any changes are proposed to the protocol. Please submit a completion report at the conclusion of the study.
